# Prevalence, associated risk factors; and patient and economic impact of multiple sensory impairment in a multi-ethnic elderly population in Singapore: the PIONEER study

**DOI:** 10.1186/s12889-024-18635-2

**Published:** 2024-04-22

**Authors:** Preeti Gupta, Aurora Chan, Vu Tai-Anh, Ryan E. K. Man, Eva K. Fenwick, Amudha Aravindhan, Chay Junxing, Joanne M. Wood, Alex A. Black, Jia Hui Ng, Ching-Yu Cheng, Charumathi Sabanayagam, Ecosse L. Lamoureux

**Affiliations:** 1 Singapore Eye Research Institute (SERI), The Academia, 20 College Road, Level 6, Singapore, 169856 Singapore; 2 Duke-NUS Medical School, Singapore, Singapore; 3 Centre for Vision and Eye Research, Queensland University of Technology, Brisbane, Australia; 4 Singapore General Hospital (SGH), Singapore, Singapore

**Keywords:** Vision, Hearing, Olfaction, Multiple sensory impairment, Population-based design, Older adults

## Abstract

**Background:**

To determine the prevalence, risk factors; and impact on patient health and economic outcomes across the laterality spectrum of multiple sensory impairment (MSI) in a multi-ethnic older Asian population.

**Methods:**

In this population-based study of Singaporeans aged ≥ 60 years, MSI was defined as concomitant vision (visual acuity > 0.3 logMAR), hearing (pure-tone air conduction average > 25 dB), and olfactory (score < 12 on the Sniffin’ Sticks test) impairments across the spectrum of laterality (any, unilateral, combination [of unilateral and bilateral], and bilateral).

**Results:**

Among 2,057 participants (mean ± SD 72.2 ± 0.2 years; 53.1% female), the national census-adjusted prevalence rates of any, unilateral, combination, and bilateral MSI were 20.6%, 1.2%, 12.2%, and 7.2%, respectively. Older age, male gender, low socioeconomic status (SES), and smoking (all *p* < 0.05) were independently associated with higher likelihood of any MSI. Compared to those with no sensory loss, those with MSI had significantly decreased mobility (range 5.4%-9.2%), had poor functioning (OR range 3.25–3.45) and increased healthcare costs (range 4–6 folds) across the laterality spectrum. Additionally, bilateral MSI had a significant decrease in HRQoL (5.5%, *p* = 0.012).

**Conclusions:**

MSI is a highly prevalent medical condition, with 1 in 5; and almost 1 in 10 community-dwelling older Asians having any and bilateral MSI, respectively, with a higher likelihood in men, smokers, and those with low SES. Critically, MSI has a substantial negative impact on patient health and economic outcomes across the laterality spectrum. Sensory testing is critical to detect and refer individuals with MSI for management to improve their functional independence and QoL.

Globally, age-related sensory impairments involving the visual, auditory, and olfactory pathways are very common, affecting up to two-thirds of older adults [1–4]. With the population ageing rapidly worldwide, the proportion of individuals with any sensory impairment is expected to almost double by 2050 [3, 4], which has been associated with several adverse outcomes including reduced mobility, falls, hospitalization, disability, morbidity, and mortality [1, 5–11].

While the prevalence, risk factor profiles, and impact of single and concurrent sensory impairments (dual sensory impairment; DSI) have been extensively researched worldwide [12–28], data on the concurrent impairments of three or more senses (multiple sensory impairment; MSI) are scarce. To date, there are only 4 studies which have explored the prevalence, risk factors and impact of MSI. These studies were on Caucasian populations and defined MSI using different criteria across studies, including different combination of concomitant vision, hearing, smell, touch, taste, vestibular and proprioception impairments. The risk factors for MSI studied by prior studies focused mainly on age and gender. In addition, prior studies on the impact of MSI were limited to outcomes such as dementia, depression, quality of life, and mortality [1, 8, 28, 29].

No MSI data are currently available in Asian populations, which is a significant knowledge gap due to differences in lifestyle, culture, environment, and nutritional habits; as well as differing access to and response from social and healthcare service providers, compared to western countries [25, 26]. This paucity of data is even more substantial, as Asia comprises up to 60% (~ 4.4 billion) of the world’s population, and accounts for more than half of sensory impairment cases globally [30]. In addition, no studies using clinically assessed MSI, have explored prevalence, risk factors, and impact of MSI laterality (any, unilateral, combination and bilateral) on patient health outcomes; as such, a detailed understanding of how MSI affects people across the spectrum of the condition is lacking. Finally, there are no data on the economic burden of MSI.

Against this background, the aims of this study were: (1) to determine the overall and age, gender, and ethnic-stratified prevalence of any, unilateral, combination and bilateral MSI in older community-dwelling Asians participating in the PopulatIoN HEalth and Eye Disease PRofile in Elderly Singaporeans study (PIONEER), a large, population-based cohort study of older Chinese, Malay, and Indians adults; and (2) to examine associated risk factors for MSI; (3) to study the impact of MSI on health outcomes (physical functioning, sarcopenia, frailty, falls and hospitalization) and its economic burden. We hypothesized that MSI is common among older adults, being more prevalent in men and minorities such as Indians and Malays due to ethnic differences in education, healthcare utilisation, and socio-economic status (SES) [12, 31], and are more likely to occur with increasing age. Furthermore, we expected to find several modifiable risk factors associated with MSI and that bilateral MSI will cause greater decrements in all outcomes compared to having no/unilateral/combination MSI. Such data will provide crucial information for resource allocation, structuring preventative strategies and developing better multidisciplinary integrated rehabilitative management for our ageing population.

## Methods

### Study population and design

PIONEER is a population-based study (2017–2022) investigating the clinical, biological, anthropomorphic, and psychosocial phenotypes of community-dwelling Chinese, Malay, and Indian Singaporeans aged ≥ 60 years to better understand the epidemiology, burden, and complex mechanisms associated with age-related sensory deterioration. A detailed methodology is reported elsewhere [32]. Briefly, study invitation letters were sent out to 6,377 individuals selected using an age-, gender-, and ethnicity- stratified sampling framework from a national database. These individuals were followed up by study recruitment officers in a home visit to ascertain eligibility and agreement to participate. Of the 6,377 invited, 1,015 (15.9%) were classified as ‘uncontactable’ because of invalid address(s), were unresponsive to ≥ 3 home visit attempts, and/or living in residences that were inaccessible because of security restrictions. In addition, 648 (10.2%) individuals were excluded because they were incarcerated, were residing in nursing homes or outside Singapore, or were deceased; while a further 994 (15.6%) were deemed ineligible because they were terminally ill, bedridden, or unable to follow/respond accurately to the subjective parts of clinical testing due to severe cognitive (assessed using 6 item Cognitive Impairment Test (6-CIT)) [33] or hearing impairment or muteness. Of the remaining 3,720 (69.4%) eligible individuals, 2,643 (71.05%) participated in the study, 1,054 (28.33%) refused, and 23 (0.62%) were undecided. PIONEER’s overall response rate was 71.5%. Reasons for refusal included lack of interest (*n* = 895, 84.9%) or time needed to participate in the study (*n* = 159, 15.1%). Compared to participants (*n* = 2,643), non-participants (*n* = 1,054) were older (*p* < 0.001), more likely to be female (*p* < 0.001), and Chinese (*p* < 0.001). About 54.8% of the sample was female, and 49.8%, 25.1%, 25.0% were Chinese, Indians, and Malays, respectively.

The study was conducted at the research clinic of the Singapore Eye Research Institute. All study procedures were approved by the SingHealth Centralized Institutional Review Board (CIRB, Reference #2016/3089) and its protocol adheres to the principles of the Declaration of Helsinki. Written informed consent was obtained from all participants in either Chinese, Malay, Tamil, or English.

### Assessment and definition of sensory measures

At the study clinic, all participants underwent standardized sensory examinations including comprehensive visual, auditory and olfactory assessment.

#### Vision

Distance visual acuity (VA) was measured monocularly using a logMAR (logarithm of the minimum angle of resolution; Lighthouse International, New York, NY) number chart at 4 m. Presenting distance VA (PDVA) was ascertained with participants wearing their habitual optical correction (if any), under standard photopic conditions (85 cd/m^2^). If the participants were unable to read the largest line of letters, the chart was moved to 2 m. However, if they were still unable to read any letters at 2 m, finger counting, hand movement and the ability of the eye to perceive light with a pen torch were assessed. Vision impairment (VI) was defined as PDVA worse than 20/40 (> 0.3 logMAR) in accordance with the 2019 World Health Organization (WHO) criteria for VI [34].

#### Hearing

Hearing was assessed using a portable pure tone audiometer (SHOEBOX) by a trained study coordinator in a room with minimal background noise interference. Air-conduction thresholds at 500 Hz, 1000 Hz, 2000 Hz and 4000 Hz were recorded, and pure tone average (PTA) was calculated for each ear. Hearing impairment (HI) was defined by PTA > 25 dB in accordance with WHO guidelines [35].

#### Olfaction

Olfaction was tested using the 16-item identification segment of the Sniffin’ Sticks test battery [36]. Using both nostrils, participants were asked to identify 16 different odors (e.g. coffee, shoe leather, orange) from felt pen tips impregnated with 4 ml of the odorant at intervals of 30 s between each odor presentation, and asked to choose the correct answer from four multiple-choice options. The number of correct answers was summed to provide an overall score ranging from 0 to 16. Smell disorder was defined based on normative values for the identification test as per the manufacturer guidelines [36]. Olfactory impairment (OI) was defined as a score of < 12.

### Definition of MSI

*Any MSI* was defined as the co-occurrence of all three sensory impairments i.e., any VI + any HI + OI; *unilateral MSI* as the co-presence of unilateral VI, unilateral HI and OI; *combination MSI* as unilateral VI + bilateral HI + OI or bilateral VI + unilateral HI + OI; and *bilateral MSI* as co-occurrence of bilateral VI, bilateral HI and OI. Absence of all three sensory impairments was considered as *no sensory impairment.*

### Covariables, risk factors and impact assessment and definition

Face-to-face interviews with trained interviewers fluent in English, Malay, Tamil, and Mandarin were conducted in participants’ preferred choice of language. At the interview, data on sociodemographic characteristics (e.g., age, gender, income, and education), lifestyle factors (e.g., smoking, alcohol use, and living alone), medical history (e.g., previous diagnosis of myocardial infarction, angina, stroke) and current medications were obtained. *Low socioeconomic status (SES)* was defined as having primary or lower education, and household monthly income < SGD$2000, and residing in a 1–2 room public housing flat. *Polypharmacy* was defined as taking ≥ 5 chronic disease medications after excluding supplements and vitamins [37].

*Health-related quality of life (HRQoL)* was measured using the EuroQoL-5 dimension (5L-EQ-5D) [38, 39]. Responses to the 5 dimensions, including mobility, self-care, usual activities, pain/discomfort, and anxiety/depression, were used to calculate an index score using the established UK EQ-5D tariffs ranging from -0.59 for the worse health state to 1.00 for perfect health. *Mobility and Independence* was assessed using the Singapore-validated 7-item Life Space Questionnaire (LSQ), with each item addressing a specific life space zone accessed within the past 3 days [40]. Each successive item asks about a concentrically larger area (inside and immediately outside home, neighbourhood, community, outside Singapore and outside Southeast Asian region). A ‘yes’ response is scored as 1 and ‘no’ is scored as 0 and the overall score is calculated by summing the responses to all seven items (range 0 to 7), with a higher score signifying a larger life space. *Functional status* was assessed using the 8-item Lawton Instrumental Activities of Daily Living (IADL) Scale [41], which assesses eight different domains (e.g. shopping, handling finances). Items were recoded to reflect increasing independence of the individual, with a score of 0 being the least independent and 2 being the most independent. ‘Dependent, low function’ is defined as a total Lawton score of < 16. Falls and hospitalization history was obtained via self-report. *Falls (yes/no) over the past 12 months* was quantified using an in-house questionnaire; and *hospitalization frequency and duration* over the past 6 months were determined using the modified healthcare services expenditure module that includes questions on the use of inpatient, outpatient emergency, mental health and complementary and alternative treatments, in addition to questions aimed at capturing lost work time and other indirect costs [42]. The module was also used to calculate *direct healthcare expenditure* (hospitalization and emergency department visit cost over the past 6 months; and mental health and outpatient services utilization over the past 3 months), with the costs extrapolated to annual healthcare cost.

Clinical covariates were obtained via a standardized clinical examination. Two measurements of systolic blood pressure (SBP) and diastolic blood pressure (DBP) were taken using a digital automatic BP monitor (Dinamap Pro Series DP110X-RW; GE Medical Systems Information Technologies, Inc), and a third measurement was obtained if the two previous SBP or DBP readings differed by more than 10- or 5-mm Hg, respectively. The mean of the two closest measurements was used in analyses. *Hypertension* was defined as SBP ≥ 140 mmHg, DBP ≥ 90 mmHg, self-reported use of anti-hypertensive medications, or self-reported history of physician-diagnosed hypertension. Height was measured using a wall-mounted, adjustable measuring scale, and weight was measured with a calibrated scientific weight scale. *Body mass index (BMI)* was calculated as weight in kilograms divided by height in meters squared (Wt[kg]/Ht[m]^2^). BMI was categorized as underweight (< 18.5), normal (18.5 ≤ BMI < 23), overweight (BMI ≥ 23 to 27.5), and obese (BMI > 27.5) according to the Asian cut-offs [43].

Gait speed was assessed with participants walking 4 m at their usual speed. *Low gait speed* was defined as a score of < 1.0 m/s [44]. Hand grip strength was measured three times using a digital hand dynamometer (JAMAR Plus +) in the dominant hand with the participant seated and elbow flexed at 90 degrees and a rest period of 30 s between each measurement. *Low grip strength* was defined as an average of the three readings of < 28 kg in males and < 18 kg in females [44]. Body composition and bone mineral density were measured using dual energy X-ray absorptiometry (DXA; Hologic Discovery-W; Hologic Inc, Bedford-MA). *Low muscle mass* was defined as DXA-assessed appendicular-lean-mass/height^2^ of < 7 kg/m^2^ for males and < 5.4 kg/m^2^ for females [44]. *Sarcopenia* was defined as having low muscle mass in the presence of either low grip strength or low gait speed [44]. According to the Fried frailty phenotype [45], *frailty* was defined as presence of ≥ 3 conditions (BMI < 18.5, low gait speed, low grip strength, exhaustion (score of < 10 for three questions from the vitality domain of the 12-item Short-form survey [SF-12]), low moderate to vigorous physical activity (< 150 min of moderate-vigorous physical activity per week)).

Blood samples were collected for haemoglobin A1c (HbA1c), random glucose, and total, high-density lipoprotein, low-density lipoprotein cholesterol, triglycerides, and serum creatinine measurements. *Diabetes* was defined as random glucose ≥ 11.1 mmol/L, HbA1c ≥ 6.5%, self-reported use of diabetic medication or reported history of physician-diagnosed diabetes. *Hyperlipidaemia* was defined as high levels of total cholesterol (≥ 5.2 mmol/L) and/or low levels of high-density lipoprotein (HDL) cholesterol (< 1 mmol/L in men and < 1.3 mmol/L in women), and/or self-reported use of lipid-lowering medications. *Chronic kidney disease (CKD)* was defined as an estimated glomerular filtration rate < 60 ml/min/1.73 m^2^ [46]. *Cardiovascular disease (CVD)* was defined as self-reported history of stroke, myocardial infarction or angina [47]. *Multimorbidity* was defined as having ≥ 3 systemic conditions (diabetes, hypertension, hyperlipidemia, CVD, CKD).

### Statistical analyses

Age-, gender- and ethnic-specific prevalence rates of MSI were determined by weighting individuals according to their sampling probabilities and standardizing to the 2020 Singapore Census. Pairwise comparisons of the prevalence rates within age, gender and race subgroups were conducted.

To determine the univariate association between sociodemographic, systemic, and clinical characteristics and any MSI, chi-square and t-tests tests were used for categorical and continuous variables, respectively. Independent associations of the above factors with any MSI were then analyzed using multivariable logistic regression. A backwards stepwise procedure was performed to select risk factors of any MSI in the final model, where variables leading to the largest decrease in AIC were removed one at a time until there is no further decrease in AIC from the removal of any variable. Furthermore, multinomial logistic regression models were performed in place of logistic regression where MSI was categorized into no MSI, unilateral/combination MSI and bilateral MSI. Due to the small number of participant in unilateral MSI group (*n* = 28), we merged unilateral and combination MSI into unilateral/combination MSI for risk factors and patient-centred impact analyses.

To examine the relationships between MSI and health outcomes, multiple linear regression was used for continuous outcomes, logistic regression for binary outcomes, and two-parts models for over-dispersed healthcare cost. For the two-parts models, logistic regression models were first used to determine if there were any independent associations between MSI and the likelihood of incurring healthcare expenditure; as well as a gamma generalised linear model with a log-link function to study if there were any differences in the amount spent on healthcare between those with and without MSI amongst those who have incurred a healthcare cost. Robust standard errors were used to account for heteroskedasticity. The described analyses were conducted for MSI across the spectrum of laterality as an exposure.

All statistical evaluations were made assuming a 2-sided test at the 5% level of significance. Statistical analyses were conducted using STATA version 17.0.

## Results

Of the 2,643 enrolled study participants, 2 were < 60 years old, 5 of races other than Chinese, Malay and Indian, and 579 had no smell data (as the smell test was introduced after the study commencement), leaving 2,057 participants included for this investigation. Of these, 1,397 had either single or dual sensory impairment. As such, 660 individuals were included for MSI determinants and impact analyses.

### Prevalence of MSI stratified by age, sex and ethnicity

The national census-adjusted prevalence of any, unilateral, combination, and bilateral MSI was 20.6% (95% CI: 18.6 – 22.7), 1.2% (95% CI: 0.7 – 2.0), 12.2% (95% CI: 10.5 – 14.0), and 7.2% (95% CI: 6.1 – 8.6), respectively. Except for unilateral MSI, the prevalence rates increased with increasing age (all *p*-trend < 0.001), and this trend was consistently observed across all sex and ethnic groups (Table 1).

**Table 1:** Prevalence of multiple sensory impairment across the spectrum of laterality, stratified by age, sex, and ethnicity in the PIONEER study sample (*N* = 2057)

Age group (year)	All (*N* = 2057) n (%)	Sex		Ethnicity		
		**Male** **(*N* = 929)** **n (%)**	**Female** **(*N* = 1128)** **n (%)**	**Chinese** **(*N* = 1000)** **n (%)**	**Malay** **(*N* = 583)** **n (%)**	**Indian** **(*N* = 474)** **n (%)**
**Any MSI**						
60–69	92 (11.4)	59 (16.3)	33 (6.6)	40 (11.2)	31 (12.9)	21 (10.9)
70–79	193 (25.4)	89 (28.0)	104 (23.1)	89 (24.4)	62 (34.0)	42 (27.3)
≥ 80	260 (48.3)	110 (44.7)	150 (50.6)	125 (47.8)	73 (54.1)	62 (48.7)
P-trend	< 0.001	< 0.001	< 0.001	< 0.001	< 0.001	< 0.001
Total	545 (20.6)	258 (22.9)	287 (18.5)	254 (20.4)	166 (22.3)	125 (19.7)
**Unilateral MSI**						
60–69	13 (1.3)	7 (1.8)	6 (0.8)	4 (1.1)	3 (1.2)	6 (3.1)
70–79	13 (1.3)	5 (1.4)	8 (1.3)	4 (1.1)	4 (2.3)	5 (3.3)
≥ 80	2 (0.5)	0 (0.0)	2 (0.9)	2 (0.6)	0 (0.0)	0 (0.0)
P-trend	0.44	0.22	0.75	0.62	0.80	0.23
Total	28 (1.2)	12 (1.5)	16 (1.0)	10 (1.0)	7 (1.4)	11 (2.8)
**Combination MSI**						
60–69	56 (7.5)	38 (10.6)	18 (2.4)	27 (7.6)	19 (8.0)	10 (5.3)
70–79	112 (15.2)	54 (17.9)	58 (12.9)	54 (14.9)	33 (18.0)	25 (16.1)
≥ 80	121 (25.2)	62 (26.1)	59 (24.6)	67 (25.8)	26 (19.9)	28 (22.3)
P-trend	< 0.001	< 0.001	< 0.001	< 0.001	< 0.001	< 0.001
Total	289 (12.2)	154 (14.5)	135 (10.1)	148 (12.4)	78 (11.7)	63 (10.1)
**Bilateral MSI**						
60–69	23 (2.7)	14 (3.9)	9 (1.4)	9 (2.5)	9 (3.6)	5 (2.6)
70–79	68 (8.8)	30 (8.8)	38 (8.9)	31 (8.4)	25 (1.8)	12 (7.9)
≥ 80	137 (22.6)	48 (18.6)	89 (25.1)	56 (21.3)	47 (34.2)	34 (26.4)
P-trend	< 0.001	< 0.001	< 0.001	< 0.001	< 0.001	< 0.001
Total	228 (7.2)	92 (7.0)	136 (7.4)	96 (7.0)	81 (9.3)	51 (6.8)

Data are presented as number of participants (weighted prevalence %). Weighted prevalence was calculated with sampling weights specific to each age group, gender, and ethnicity to adjust for oversampling and post-stratification weights to align to the population distribution based on the 2020 Singapore Census

*Abbreviations*: *MSI* multiple sensory impairments

Sex-stratified results revealed that males had higher prevalence rates of any, unilateral and combination MSI than females, both overall (22.9% in males vs 18.5% in females for any MSI, 1.5% in males vs 1% in females for unilateral MSI, and 14.5% in males vs 10.1% in females for combination MSI), and across age groups, with the exception of the ≥ 80 year age bracket for any MSI, while the prevalence of bilateral MSI was higher in females (overall 7.4% in women vs 7% in men). However, these differences were only statistically significant for any and combination MSI (all *p* < 0.05; pairwise comparison, data not shown).

For ethnicity-stratified results, Malays had a higher prevalence of any (22.3% in Malays, 20.4% in Chinese and 19.7% in Indians) and bilateral MSI (9.3% in Malays, 7.0% in Chinese and 6.8% in Indians), while Indians had a higher prevalence of unilateral MSI (2.8% in Indians, 1.4% in Malays and 1% in Chinese) and Chinese had a higher prevalence of combination MSI (12.4% in Chinese, 11.7% in Malays and 10.1% in Indians). However, these differences were not significant (all *p* > 0.05; pairwise comparison, data not shown).

### Sociodemographic characteristics of participants with MSI

Of the 660 assessed individuals, 115 (17.4%) and 545 (82.6%) had no sensory impairment and MSI, respectively (Table 2). Compared to those with no sensory impairment, participants with any MSI were older, of male gender, had low SES, were living alone, and a greater proportion had systemic conditions (diabetes, hypertension, CVD, and CKD), multimorbidity, and polypharmacy (all *p* < 0.05; Table 2).

**Table 2:** Comparison of characteristics of participants with no sensory impairment and any multiple sensory impairment in the PIONEER study population

	**Overall** **(*N* = 660)**	**None** **(*N* = 115)**	**Any MSI** **(*N* = 545)**	**P**^a^
**Age group**				**< 0.001**
60–69	187 (28.3)	95 (82.6)	92 (16.9)	
70–79	211 (32.0)	18 (15.7)	193 (35.4)	
≥ 80	262 (39.7)	2 (1.7)	260 (47.7)	
**Sex** (female)	367 (55.6)	80 (69.6)	287 (52.7)	**< 0.001**
**Race**				0.425
Chinese	312 (47.3)	58 (50.4)	254 (46.6)	
Malay	194 (29.4)	28 (24.4)	166 (30.5)	
Indian	154 (23.3)	29 (25.2)	125 (22.9)	
**Low SES**	132 (26.0)	5 (4.6)	127 (31.8)	**< 0.001**
**Living Alone**	65 (10.2)	5 (4.4)	60 (11.5)	**0.025**
**Systemic Conditions**				
Diabetes	229 (38.5)	29 (27.1)	200 (41.0)	**0.008**
Hypertension	575 (87.4)	87 (75.7)	488 (89.9)	**< 0.001**
Dyslipidemia	530 (86.3)	100 (90.9)	430 (85.3)	0.122
CVD	111 (20.6)	9 (8.3)	102 (23.7)	**< 0.001**
CKD	160 (26.9)	1 (0.9)	159 (32.7)	**< 0.001**
**BMI**				0.782
BMI < 18.5	34 (5.2)	7 (6.1)	27 (4.9)	
18.5 ≤ BMI < 23	204 (30.9)	33 (28.7)	171 (31.4)	
BMI > 23	422 (63.9)	75 (65.2)	347 (63.7)	
**Multimorbidity**	253 (46.2)	28 (27.2)	225 (50.6)	**< 0.001**
**Polypharmacy**	141 (22.2)	11 (9.7)	130 (24.9)	**< 0.001**
**Smoking**				0.35
Never or past smoker	583 (91.7)	107 (93.9)	476 (91.2)	
Current Smoker	53 (8.3)	7 (6.1)	46 (8.8)	
**Alcohol frequency**				0.224
None	564 (91.0)	90 (87.4)	474 (91.7)	
≤ 4 days per week	42 (6.8)	11 (10.7)	31 (6.0)	
> 4 days per week	14 (2.3)	2 (1.9)	12 (2.3)	

Data presented as number (weighted proportions). Weighted proportions were calculated with sampling weights specific to each age group, gender, and ethnicity to adjust for oversampling

*Abbreviations*: *MSI* multiple sensory impairments, *SES* socioeconomic status, *CVD* cardiovascular disease, *CKD* chronic kidney disease, *BMI* body mass index

^a^*P*-values were calculated using chi-square test or Fisher exact test for categorical variables, as appropriate, and t-test for continuous variables

### Risk factors associated with MSI

In multivariable models exploring the factors associated with any MSI (Table 3), we found that older age (per year increase: odds ratio [OR]: 1.33, 95% confidence interval [CI]: 1.25, 1.42; *p* < 0.001); male gender (OR: 2.84; 95% CI: 1.43, 5.65; *p* = 0.003), low SES (OR: 6.15; 95% CI: 2.06, 18.15; *p* = 0.001), and smoking (OR: 4.91, 95% CI: 1.60,15.01; *p* = 0.005) were independently associated with higher odds of any MSI. In contrast, we observed borderline significant association between low-moderate levels of weekly alcohol consumption (≤ 4 days per week compared to no alcohol consumption at all; OR: 0.35, 95% CI: 0.12, 1.02; *p* = 0.054) and lower odds of any MSI. Furthermore, for MSI laterality, including unilateral/combination or bilateral MSI, similar risk factors were observed (data not shown).

**Table 3:** Factors associated with any multiple sensory impairment

Risk Factors	Odds Ratio (95% Confidence Interval)			
	**Unadjusted Model**	**Full Model**	**Final Model**^a^	***P*-value**^b^
**Age**	1.30 (1.24, 1.37)	1.33 (1.24, 1.43)	1.33 (1.25, 1.42)	**< 0.001**
**Sex** (Male)	2.05 (1.33, 3.16)	2.41 (1.15, 5.05)	2.84 (1.43, 5.65)	**0.003**
**Race**				
Chinese	Reference	Reference	-	
Malay	1.34 (1.24, 1.45)	1.37 (0.58, 3.21)	-	
Indian	1.77 (0.81, 3.89)	1.16 (0.50, 2.69)	-	
**Low SES**	9.71 (3.86, 24.41)	8.21 (2.31, 29.22)	6.15 (2.06, 18.35)	**0.001**
**Living alone**	2.81 (1.10, 7.15)	1.07 (0.29, 3.90)	-	
**BMI**				
BMI < 18.5	0.74 (0.30, 1.85)	0.50 (0.10, 2.45)	-	
18.5 ≤ BMI < 23	Reference	Reference	-	
BMI ≥ 23	0.89 (0.57, 1.40)	0.94 (0.43, 2.07)	-	
**Multimorbidity**	2.74 (1.71, 4.39)	0.91 (0.44, 1.86)	-	
**Polypharmacy**	3.07 (1.60, 5.89)	1.50 (0.57, 3.92)	-	
**Current smoker**	1.48 (0.65, 3.36)	5.85 (1.66, 20.67)	4.91 (1.60, 15.01)	**0.005**
**Alcohol frequency**				
None	Reference	Reference	Reference	
≤ 4 days per week	0.54 (0.26, 1.10)	0.26 (0.08, 0.85)	0.35 (0.12, 1.02)	0.054
> 4 days per week	1.14 (0.25, 5.18)	3.25 (0.55, 19.37)	2.77 (0.50, 15.46)	0.246

*Abbreviations*: *SES* socioeconomic status, *BMI* body mass index

^a^Final model was obtained using backwards stepwise procedure where variable which contributed to the greatest decrease in AIC was removed one at a time until further removal does not result in a decrease in AIC

^b^*P*-values were calculated from Wald test

### Patient-centred impact and economic burden of MSI

Compared to those with no sensory impairment, those with any MSI had significantly larger reductions in life space mobility (6.8%; *p* = 0.012) and greater odds of being dependent i.e., low function (OR: 3.31; 95% CI: 1.18, 9.32, *p* = 0.023; Table 4). Furthermore, including unilateral/combination and bilateral MSI, compared to participants with no sensory impairment, participants with unilateral/combination MSI had a 5.4% reduction in independent mobility (β: -0.24; 95% CI: -0.47, -0.01, *p* = 0.04) while those with bilateral MSI reported a 10.8% reduction (β: -0.46; 95% CI: -0.78, -0.13, *p* = 0.006; Table 5). Similarly, individuals with unilateral/combination (OR: 3.25; 95% CI: 1.15, 9.19; *p* = 0.026) and bilateral MSI (OR: 3.45; 95% CI: 1.13, 10.59; *p* = 0.03; Table 5) also had increased odds of low IADL. However, compared to participants with no sensory impairment, only those with bilateral MSI had significant reductions in HRQoL (5.5%; β: -0.05; 95% CI: -0.09, -0.01, *p* = 0.012). No significant associations between MSI and outcomes such as sarcopenia, frailty, falls, and hospitalization were observed. Additionally, among those who spent on healthcare, healthcare expenditure for those with MSI (exp(β): 4.67; 95% CI: 2.21, 9.85, *p* < 0.001) was more than four times those without sensory impairment (Table 6); while those with unilateral/combination (exp(β): 4.19; 95% CI: 1.95, 9.01, *p* < 0.001) and bilateral (exp(β): 6.18; 95% CI: 2.04, 18.73, *p* = 0.001) MSI had 4- or 6-fold increased healthcare cost compared to those with no sensory impairment (Table 7).

**Table 4:** Associations between any multiple sensory impairments and patient-centered outcomes

Outcomes	Exposure	Coefficient (95% CI)^a^	*P*-value^b^	Change (%)
**HRQoL**	None	Reference		
	Any MSI	β: -0.03 (-0.06, 0.00)	0.059	-3
**Life space mobility**	None	Reference		
	Any MSI	β: -0.30 (-0.53, -0.07)	**0.012**	-6.8
**Low IADL**	None	Reference		
	Any MSI	OR: 3.31 (1.18, 9.32)	**0.023**	53.8
**Falls**	None	Reference		
	Any MSI	OR: 0.67 (0.33, 1.36)	0.271	-33.2
**Hospitalization**	None	Reference		
	Any MSI	OR: 2.55 (0.38, 17.04)	0.334	55.7
**Sarcopenia**	None	Reference		
	Any MSI	OR: 2.21 (0.99, 4.93)	0.054	26.2
**Frailty**	None	Reference		
	Any MSI	OR: 1.43 (0.72, 2.83)	0.311	12.4

β(s) were from normal regression models and ORs were from logistic regression models

*Abbreviations*: *MSI* multiple sensory impairments, *HRQoL* health-related quality of life, *IADL* instrumental activities of daily living, *OR* odds ratio, *CI* confidence interval

^a^Adjusted for age, gender, race, socioeconomic status, living alone, smoking status, alcohol frequency, body mass index, diabetes, hypertension, and polypharmacy

^b^*P*-values were calculated from Wald test

**Table 5:** Associations between laterality of multiple sensory impairments and patient-centered outcomes

**Outcomes**	**Exposure**	**Coefficient (95% CI)**^***a***^	***P*-value**^b^	**Change (%)**
**HRQoL**	None	Reference		
	Unilateral/Combination MSI	β: -0.02 (-0.05, 0.01)	0.178	-2.1
	Bilateral MSI	β: -0.05 (-0.09, -0.01)	**0.012**	-5.5
**Life space mobility**	None	Reference		
	Unilateral/Combination MSI	β: -0.24 (-0.47, -0.01)	**0.044**	-5.4
	Bilateral MSI	β: -0.46 (-0.78, -0.13)	**0.006**	-10.8
**Low IADL**	None	Reference		
	Unilateral/Combination MSI	OR: 3.25 (1.15, 9.19)	**0.026**	53.4
	Bilateral MSI	OR: 3.45 (1.13, 10.59)	**0.03**	55
**Falls**	None	Reference		
	Unilateral/Combination MSI	OR: 0.66 (0.32, 1.35)	0.255	-35.4
	Bilateral MSI	OR: 0.71 (0.30, 1.66)	0.431	-27.8
**Hospitalization**	None	Reference		
	Unilateral/Combination MSI	OR: 2.14 (0.31, 15.03)	0.444	48.8
	Bilateral MSI	OR: 3.64 (0.44, 30.19)	0.231	66.7
**Sarcopenia**	None	Reference		
	Unilateral/Combination MSI	OR: 2.27 (1.00, 5.14)	0.051	26.8
	Bilateral MSI	OR: 2.07 (0.85, 5.04)	0.11	24.5
**Frailty**	None	Reference		
	Unilateral/Combination MSI	OR: 1.28 (0.63, 2.59)	0.499	9
	Bilateral MSI	OR: 1.81 (0.84, 3.91)	0.132	19.2

β(s) were from normal regression models and ORs were from logistic regression models

*Abbreviations*: *MSI* multiple sensory impairments, *HRQoL* health-related quality of life, *IADL* instrumental activities of daily living, *OR* odds ratio, *CI* confidence interval

^a^Adjusted for age, gender, race, socioeconomic status, living alone, smoking status, alcohol frequency, body mass index, diabetes, hypertension, and polypharmacy

^b^*P*-values were calculated from Wald test

**Table 6:** Associations between any multiple sensory impairments and healthcare cost

**Exposure**	**OR (95% CI)**^a^	***P*-value**^b^	**exp(β) (95% CI)**^a^	***P*-value**^b^
None	Reference		Reference	
Any MSI	0.71 (0.37, 1.35)	0.30	4.67 (2.21, 9.85)	< 0.001

OR was from logistic regression models and exp(β) was from gamma generalised linear models with a log-link function as part of two-parts model. Robust standard errors were used for all models

*Abbreviations*: *MSI* multiple sensory impairments, *OR* odds ratio, *CI* confidence interval

^a^Adjusted for age, gender, race, socioeconomic status, living alone, smoking status, alcohol frequency, body mass index, diabetes, hypertension, and polypharmacy

^b^*P*-values were calculated from Wald test

**Table 7:** Associations between laterality of multiple sensory impairments and healthcare cost

**Exposure**	**OR (95% CI)**^a^	***P*-value**^b^	**exp(β) (95% CI)**^a^	***P*-value**^b^
None	Reference		Reference	
Unilateral/Combination MSI	0.74 (0.39, 1.43)	0.372	4.19 (1.95, 9.01)	< 0.001
Bilateral MSI	0.64 (0.30, 1.34)	0.234	6.18 (2.04, 18.73)	0.001

OR was from logistic regression models and exp(β) was from gamma generalised linear models with a log-link function as part of two-parts model. Robust standard errors were used for all models

*Abbreviations*: *MSI* multiple sensory impairments, *OR* odds ratio, *CI* confidence interval

^a^Adjusted for age, gender, race, socioeconomic status, living alone, smoking status, alcohol frequency, body mass index, diabetes, hypertension, and polypharmacy

^b^*P*-values were calculated from Wald test

## Discussion

In our large, contemporary, population-based study of multi-ethnic older Asian adults living in Singapore, almost 21%, 1.2% 12.2% and 7.2% of older Singaporeans had any, unilateral, combination and bilateral MSI, respectively. Older age, male gender, low SES, and smoking were the main risk factors, while low to moderate alcohol consumption was associated with lower likelihood of MSI. We found adverse effects on independent mobility, functioning and healthcare expenditure across the spectrum of MSI laterality, while HRQoL was affected only in those with bilateral MSI. These findings provide much needed evidence that MSI is a significant health concern in Asia and emphasize the urgent need to incorporate routine multiple sensory screening (vision, hearing and smell) in basic medical assessments for older individuals, so that early detection and interventions can be adopted to prevent, slow, or even reverse the development and progression of MSI to maintain functional independence and decrease economic burden in this growing segment of the population.

Ours is the first population-based study to report on the prevalence of MSI across the spectrum of laterality, making it difficult to compare our results. However, our prevalence estimates of bilateral MSI are similar to the bilateral prevalence of combined vision, hearing and olfactory impairment assessed objectively in the Atherosclerosis Risk in Communities (ARIC; 8.1%) and Baltimore Longitudinal Study of Aging (BLAS; 5.2%) studies in community-dwelling older adults in the United States [48]; higher than the self-reported MSI prevalence of 3% in the English Longitudinal Study of Aging (ELAS) of community-dwelling adults aged 52 years and older [29]; but lower than the 26% objectively assessed MSI (although defined as concomitant vision, hearing, smell and taste impairment) prevalence reported in black and white participants aged 70–79 years from the Health ABC study [8]. These result inconsistencies may be explained by differences in how sensory functions were assessed (self-report or objectively defined), their combinations such as co-occurrence of vision, hearing, smell or taste impairment to define MSI, and the different thresholds used to define the individual impairments. This could also be due to differences in study characteristics, such as ethnicity, lifestyle, culture, access to healthcare and participants’ age range, which is important as sensory functions decline with advanced age [1].

In our study, the highest prevalence of MSI across the spectrum of laterality (except unilateral MSI) was observed in the oldest age group, and this finding persisted in multivariable regression models. While these results align with age-related trends reported in other studies in Western populations, MSI was differently defined. For example, in the study by Correia and associates of older adults in the United States, with MSI defined as concomitant vision, hearing, smell, touch and taste impairment [1], and the ARIC and BLAS studies in community-dwelling adults in the US, with MSI defined as concomitant impairments in vision, hearing, smell, vestibular and proprioception [48], have all found a significantly higher prevalence of MSI as age increases.

To date, no study has explored the risk factors of MSI, warranting an urgent need for future studies to elicit a better understanding of the MSI risk factors. Our finding that lower SES was associated with MSI is unsurprising, as individuals with lower SES levels are more likely to be employed in blue collar jobs with greater potential for occupational loud noise and constant sunlight exposure that predispose them to developing hearing and vision loss [49]. Furthermore, older adults with lower SES are less likely to utilize healthcare services and encounter difficulties with regards to healthcare access [49–51]. However, it is important to note that the confidence intervals for our odds ratio were very wide due to the small number of participants with these exposures in the no sensory impairment group and, as such, our results must be interpreted with caution. Interestingly, we observed borderline significant association between low-moderate alcohol consumption and lower risk of MSI, while high consumption predisposed to greater MSI risk (although insignificant). Low-moderate alcohol consumption may have a positive effect on metabolic profiles (e.g., blood pressure, lipid and glycemic levels), leading to decreased cardiovascular risk factors, enhanced neurosensory protection, and consequently lower likelihood of MSI [52]. Our borderline significant results warrants further cohort studies to explore the potential effect of alcohol consumption on MSI.

Our finding of a substantial negative impact of MSI on HRQoL, independent mobility and functioning corroborate limited reported findings in the Western populations. For instance, in ELAS, self-reported MSI was associated with poorer QoL and greater risk of depressive symptoms [29]. Brenowitz and colleagues showed that the presence of MSI was associated with increased risk of dementia in older US adults (70–79 years of age) [8]. However, due to low number of individuals with MSI and depression, loneliness, and cognitive impairment in our sample, we were unable to explore the impact of MSI on these outcomes. We also did not observe any significant associations of MSI with an increased likelihood of sarcopenia, frailty, falls and hospitalization, possibly because of lack of statistical power related to the small number of people with these outcomes, particularly in no sensory impairment group. Studies with larger samples are needed to elicit a better understanding of the MSI-health and patient-reported outcomes relationship.

Based on the indirect causal pathway [1, 8, 28, 29] and some of our own findings, we speculate that MSI, particularly impairments in hearing and vision, may lead to social isolation, depression, poor nutrition, reduced physical activity, and functional limitations which may, in turn, lead to frailty, accelerated cognitive decline and poor QoL. Given that sensory impairments can be preventable or at least delayed, early detection and intervention for hearing (hearing aids) and vision (e.g. surgical removal of cataract, provision of glasses), and to a lesser extent smell (olfactory training) loss might help to alleviate loneliness and maintain positive well-being in later life by not restricting social participation which, in turn, can help decrease the risk of future frailty, cognitive impairment and functional dependence, potentially improving their QoL and economic well-being [53–56].

Importantly, we found that among those who spent on healthcare, healthcare expenditure for those with MSI across the spectrum of laterality was 4- to 6-times those without sensory loss. However, no study to date has evaluated MSI contribution to healthcare spending, as such, cohort studies are needed to determine healthcare utilization and economic burden associated with MSI so that greater efforts to reduce healthcare expenditure such as subsidizing the cost of sensory treatments in those with MSI can be instituted. Overall, our findings emphasize the need for strategies focusing on detecting and treating MSI to minimize the loss of functional independence, mobility and QoL, and economic burden.

Strengths of this study include a large, geographically representative, ethnically diverse, and extremely well-characterized older cohort, which means our findings are likely to be generalizable to the older Singaporean community; the availability of high-quality objectively assessed sensory and systemic data; and a rich collection of bio-samples that enabled us to adjust for many relevant covariables. However, some limitations must also be noted. Data on the sense of taste and touch have not been collected, restricting our analyses to the remaining three senses. Audiometric testing was conducted in a non-soundproof room where the background noise might be above the maximum recommended limit [57], which may have led to over-estimation of hearing impairment in our population. Certain data, such as participants’ medical history and history of falls, were self-reported as we were unable to access electronic medical records for verification due to Singapore’s strict data protection laws. This could also be a key reason why we did not find any significant associations between MSI and outcomes such as falls or hospitalization in our study. Studies with objectively assessed medical conditions are needed to elicit a better understanding of MSI with these outcomes. Moreover, we did not conduct any cultural adaptation of the Sniffin’ Sticks test for our older Asian population, suggesting that our results may need to be interpreted with caution. Studies are needed to culturally validate and establish population norms for more precise assessments of olfactory function in Singapore. In addition, we excluded individuals with dementia, severe deafness and/or muteness from our study due to their inability to follow/respond accurately to the subjective parts of clinical testing; hence our results are not generalizable to these population subgroups. The EQ5D, being a generic HRQoL questionnaire, may be insensitive to the person-centred impact of specific sensory impairments and/or their associated interventions, which potentially explains our non-significant findings, particularly for unilateral/combination MSI. The difficulty in assessing such patient-reported difficulties with currently available generic HRQoL questionnaires advocate for the development and validation of specific patient-reported outcome measures (PROMs) to more precisely quantify the impact of sensory deterioration on QoL, possibly leading to better tailored intervention strategies. Lastly, our cross-sectional design limits our ability to infer the directionality of the relationships between potential risk factors and the impact of MSI, and to determine whether the observed relationships change over time. Longitudinal studies are needed to better understand causality. Indeed, our group has recently (Nov 2022) commenced a 4.5-year follow-up visit for the PIONEER study (PIONEER-II), which will help us understand if the presence of multiple sensory deficits is a clinical biomarker identifying older adults at high risk of poor health outcomes who could be targeted for early intervention to prevent the onset of adverse outcomes.

In conclusion, 1 in 5; and almost 1 in 10 community-dwelling older Asians have any and bilateral MSI, respectively, with a higher likelihood in older adults, males, smokers, and those with low SES. MSI has a substantial negative impact on health and economic outcomes across the spectrum of laterality. These findings highlight the importance of including tests of multiple sensory functions in routine assessments to prevent the onset of MSI. Clinically, preventing progression from unilateral to bilateral impairment or mitigating the effects of MSI using interventions like glasses, hearing aids and olfactory training is important to improve functional independence and HRQoL, thereby reducing their economic burden. Such efforts will have a large public health effect and can contribute to interventions, program delivery, and policy refinements to improve sensory health outcomes in older adults, enabling them to maintain good functional health and live independently for longer.

## Data Availability

All data generated or analyzed during this study are included in this article.
